# Abnormal cleavage up to Day 3 does not compromise live birth and neonatal outcomes of embryos that have achieved full blastulation: a retrospective cohort study

**DOI:** 10.1093/humrep/deae062

**Published:** 2024-03-29

**Authors:** Tammy Lee, Kelli Peirce, Jay Natalwala, Vincent Chapple, Peter J Mark, Katherine Sanders, Yanhe Liu

**Affiliations:** School of Human Sciences, The University of Western Australia, Crawley, WA, Australia; Fertility North, Joondalup Private Hospital, Joondalup, WA, Australia; Fertility North, Joondalup Private Hospital, Joondalup, WA, Australia; Fertility North, Joondalup Private Hospital, Joondalup, WA, Australia; Fertility North, Joondalup Private Hospital, Joondalup, WA, Australia; School of Human Sciences, The University of Western Australia, Crawley, WA, Australia; School of Human Sciences, The University of Western Australia, Crawley, WA, Australia; School of Human Sciences, The University of Western Australia, Crawley, WA, Australia; Fertility North, Joondalup Private Hospital, Joondalup, WA, Australia; School of Medical and Health Sciences, Edith Cowan University, Joondalup, WA, Australia; School of Health Sciences and Medicine, Bond University, Robina, QLD, Australia

**Keywords:** direct cleavage, reverse cleavage, <6 intercellular contact points, blastulation, live birth

## Abstract

**STUDY QUESTION:**

Do embryos displaying abnormal cleavage (ABNCL) up to Day 3 have compromised live birth rates and neonatal outcomes if full blastulation has been achieved prior to transfer?

**SUMMARY ANSWER:**

ABNCL is associated with reduced full blastulation rates but does not impact live birth rates and neonatal outcomes once full blastulation has been achieved.

**WHAT IS KNOWN ALREADY?:**

It is widely accepted that ABNCL is associated with reduced implantation rates of embryos when transferred at the cleavage stage. However, evidence is scarce in the literature reporting birth outcomes from blastocysts arising from ABNCL embryos, likely because they are ranked low priority for transfer.

**STUDY DESIGN, SIZE, DURATION:**

This retrospective cohort study included 1562 consecutive autologous *in vitro* fertilization cycles (maternal age 35.1 ± 4.7 years) performed at Fertility North, Australia between January 2017 and June 2022. Fresh transfers were performed on Day 3 or 5, with remaining embryos cultured up to Day 6 before vitrification. A total of 6019 embryos were subject to blastocyst culture, and a subset of 664 resulting frozen blastocysts was included for live birth and neonatal outcome analyses following single transfers.

**PARTICIPANTS/MATERIALS, SETTING, METHODS:**

ABNCL events were annotated from the first mitotic division up to Day 3, including direct cleavage (DC), reverse cleavage (RC) and <6 intercellular contact points at the 4-cell stage (<6ICCP). For DC and RC in combination, the ratios of affected blastomeres over the total number of all blastomeres up to Day 3 were also recorded. All pregnancies were followed up until birth with gestational age, birthweight, and sex of the baby being recorded.

**MAIN RESULTS AND THE ROLE OF CHANCE:**

Full blastulation rates for embryos showing DC (19.5%), RC (41.7%), <6ICCP (58.8%), and mixed (≥2) ABNCL types (26.4%) were lower than the rates for those without ABNCL (67.2%, *P *<* *0.01 respectively). Subgroup analysis showed declining full blastulation rates with increasing ratios of combined DC/RC affected blastomeres over all blastomeres up to the 8-cell stage (66.2% when 0 affected, 47.0% when 0.25 affected, 27.4% when 0.5 affected, 14.5% when 0.75 affected, and 7.7% when all affected, *P *<* *0.01). However, once full blastulation had been achieved, no difference was detected between DC, RC, <6ICCP, and no ABNCL blastocysts following single frozen transfers in subsequent live birth rates (25.9%, 33.0%, 36.0% versus 30.8%, *P *>* *0.05, respectively), gestational age (38.7 ± 1.6, 38.5 ± 1.2, 38.3 ± 3.5 versus 38.5 ± 1.8 weeks, *P *>* *0.05, respectively) and birthweight (3343.0 ± 649.1, 3378.2 ± 538.4, 3352.6 ± 841.3 versus 3313.9 ± 509.6 g, *P *>* *0.05, respectively). Multiple regression (logistic or linear as appropriate) confirmed no differences in all of the above measures after accounting for potential confounders.

**LIMITATIONS, REASONS FOR CAUTION:**

Our study is limited by its retrospective nature, making it impossible to control every known or unknown confounder. Embryos in our dataset, being surplus after selection for fresh transfer, may not represent the general embryo population.

**WIDER IMPLICATIONS OF THE FINDINGS:**

Our findings highlight the incremental impact of ABNCL, depending on the ratio of affected blastomeres up to Day 3, on subsequent full blastulation. The reassuring live birth and neonatal outcomes of ABNCL blastocysts imply a potential self-correction mechanism among those embryos reaching the blastocyst stage, which provides valuable guidance for clinical practice and patient counseling.

**STUDY FUNDING/COMPETTING INTEREST(S):**

This research is supported by an Australian Government Research Training Program (RTP) Scholarship. All authors report no conflict of interest.

**TRIAL REGISTRATION NUMBER:**

N/A.

## Introduction

Embryo selection is a critical step in the *in vitro* fertilization (IVF) process to diminish time to pregnancy, as well as for reducing psychological stress and costs to the patient. A shift to single embryo transfer has been gaining a wider global consensus to minimize multiple pregnancies (Practice Committee of the American Society for Reproductive Medicine and the Practice Committee for the Society for Assisted Reproductive Technologies, 2021), consequently decreasing a myriad of adverse complications associated with multiple births, such as mortality and morbidity (D’Antonio *et al.*, 2013), shorter gestational age (Blondel *et al.*, 2006), low birthweight (Giorgione *et al.*, 2021), and long-term health outcomes (Wainstock *et al.*, 2023). However, selection of the embryo with the best implantation potential by non-invasive approaches remains a challenge (Gardner *et al.*, 2015; Glatstein *et al.*, 2023).

Time-lapse incubation (TLI) allows uninterrupted culture and continuous monitoring of embryos. This offers new promise for better selection compared to traditional static morphological assessment (Alpha Scientists in Reproductive Medicine and ESHRE Special Interest Group of Embryology, 2011) and extended culture to the Day 5 or 6 blastocyst stage (Gardner and Schoolcraft, 1999). Many TLI embryo selection algorithms utilize both qualitative and quantitative measures to select an embryo with the highest implantation potential (Meseguer *et al.*, 2011; Liu *et al.*, 2016; Petersen *et al.*, 2016). These quantitative and qualitative parameters can be annotated in TLI but are otherwise missed in standard benchtop incubation. Quantitative parameters, such as embryo morphokinetics, include times to reach a range of biological milestones such as pronuclear appearance, 2-cell stage, and start of blastulation (Ciray *et al.*, 2014). Qualitative traits include morphometric features as early as the pronuclear stage (Orevich *et al.*, 2022), spontaneous collapsing at the blastocyst stage (Bickendorf *et al.*, 2023), and abnormal cleavage (ABNCL) patterns at the cleavage stage such as direct cleavage (DC) (Rubio *et al.*, 2012; Ciray *et al.*, 2014), reverse cleavage (RC) (Liu *et al.*, 2014) and less than six intracellular contact points at the 4-cell stage (<6ICCP) (Liu *et al.*, 2015). A recent meta-analysis has highlighted the preferable inter-laboratory transferability of qualitative parameters over quantitative parameters (Liu *et al.*, 2020), potentially due to the varying embryo morphokinetics in response to different culture conditions (Kasterstein *et al.*, 2013) and patient populations (Fréour *et al.*, 2013).

Unlike other categories of qualitative parameters used for TLI embryo selection, ABNCL patterns are relatively better defined and clinically applied (ESHRE Working group on Time-lapse technology, 2020). It is well reported that ABNCL leads to a myriad of adverse outcomes such as reduced blastulation (Meseguer *et al.*, 2011; Athayde Wirka *et al.*, 2014; Shavit *et al.*, 2023), lower blastocyst quality (Ho *et al.*, 2018; Jin *et al.*, 2022), and decreased implantation potential when transferring at the cleavage stage (Rubio *et al.*, 2012; Liu *et al.*, 2014, 2015; Fan *et al.*, 2016; Barrie *et al.*, 2017). Previous studies have focused on the incidence of DC at the 1-cell and 2-cell stage (Athayde Wirka *et al.*, 2014; Fan *et al.*, 2016), while few have considered the combined impacts of different ABNCL types up to Day 3 or beyond the 4-cell stage by a structured classification system. Moreover, the role of blastulation in embryos displaying ABNCL remain underexplored, with a lack of live birth and neonatal outcome data in the literature. Therefore, the current study aims to: (i) quantify the combined impacts of ABNCL on full blastulation, according to the ratio of affected blastomeres over total blastomeres up to Day 3, and (ii) evaluate live birth rates and neonatal outcomes of ABNCL embryos that have successfully blastulated.

## Materials and methods

This retrospective cohort study included 1562 consecutive autologous IVF and intra-cytoplasmic sperm injection (ICSI) cycles (maternal age 35.1 ± 4.7 years) performed at Fertility North (Perth, Australia) between January 2017 and June 2022. All included cycles led to either a Day 3 or 5 fresh transfer or freezing of all embryos as decided by the treating clinician, with all remaining embryos being cultured up to Day 6. Live birth outcomes and neonatal analyses were conducted based on a subset of 664 resulting blastocysts which had subsequently undergone a single frozen transfer. Table 1 describes baseline characteristics of cycles included in the study. Retrospective data analysis was approved by the Ramsay Health Care Human Research Ethics Committee (2022/ETH/0073) and the Human Research Ethics Office of The University of Western Australia (2023/ET000715).

**Table 1.** Cycle characteristics.

Characteristic	Value
Number of oocyte collection cycles (n)	1562
Female age at oocyte collection (years, mean ± SD and range)	35.1 ± 4.7 21.4–48.1
IVF/ICSI ratio	636/926
Number of fertilized oocytes (n)	9451
Number of Day 3 embryos subject to blastocyst culture (n)	6019
Number of blastocysts formed (n)	3239
Number of single frozen blastocyst transfers (n)	664
Female age at oocyte collection (years, mean ± SD and range)	34.0 ± 4.4 23.8–45.7
Body mass index (mean ± SD and range)	25.5 ± 4.8 17.2–39.1
IVF/ICSI ratio	241/423
Sperm type (partner/donor)	512/152
Number of Day 5 blastocysts (n)	486
Number of Day 6 blastocysts (n)	178
Number of single frozen blastocyst transfer cycles leading to a live birth (n/%)	208 (31.3%)
Live birth babies included for analysis (n)*	204
Baby sex ratio (male/female)	97/107
Gestational age (weeks, mean ± SD)	38.5 ± 1.9
Birthweight (grams, mean ± SD)	3327.4 ± 550.4
Number of preterm deliveries (<38 weeks, n/%)	24 (11.8%)
Number of low birthweight (<2500 g, n/%)	9 (4.4%)

* Note: Four cycles were excluded from the neonatal analysis, including one with monozygotic twins and three with unknown birthweight outcomes.

### Gamete preparation, fertilization, and embryo culture

Ovarian stimulation, transvaginal oocyte aspiration, sperm preparation (either partner or donor), and insemination via either conventional IVF or ICSI were performed according to previous publication (Liu *et al.*, 2014). Oocyte-cumulus complexes (OCCs) were washed and held in G-IVF™ Plus media (Vitrolife, Sweden) at 6% CO_2_ and 37°C until completion of oocyte collection. OCCs were then transferred to wells containing G-IVF™ Plus media (Vitrolife) overlaid with Ovoil™ (Vitrolife) at 6% CO_2_, 5% O_2_, and 89% N_2_ at 37°C for culture before conventional IVF insemination or denudation for ICSI.

Oocytes for insemination by ICSI were denuded by brief exposure to SynVitro™ Hyadase (Origio, Denmark) for a maximum of 10 s, followed by mechanical removal of cumulus cells in G-MOPS™ (Vitrolife). Oocytes at the metaphase II stage were injected with a single spermatozoon and subsequently loaded into the EmbryoScope+ incubator (Vitrolife) for culture at 6% CO_2_, 5% O_2_, and 89% N_2_ at 37°C. From July 2020, the laboratory shifted from a sequential (G1-Plus™/G2-Plus™ with a changeover on Day 3) to a single-step (G-TL™ Plus up to Day 6 without refresh) culture strategy. For conventional IVF, prepared sperm were mixed with OCCs for overnight co-incubation, before fertilization was assessed in 16–18 h. Oocytes with confirmed two pronuclei were then placed into the EmbryoScope+ for culture up to Day 6.

### Time-lapse annotation for ABNCL events

A total of 9451 fertilized oocytes were cultured up to Day 3. Amongst these embryos, 3432 were either transferred on Day 3 or classified as grade F, the lowest grade, according to our previously published algorithm (Liu *et al.*, 2016), and were subsequently excluded from analysis. The remaining 6019 embryos were fully annotated for morphokinetic features and subjected to blastocyst culture.

Three ABNCL events, DC, RC, and <6ICCP, were manually annotated up to Day 3 with the EmbryoViewer^®^ software (Vitrolife). We defined DC as the division of one blastomere into three daughter blastomeres at any stage, previously termed trichotomous mitosis by Ciray *et al.* (2014). RC was defined as either type I: blastomeric fusion following cell division, or type II: failed cytokinesis after karyokinesis (Liu *et al.*, 2014). The ABNCL event <6ICCP was classified if there were fewer than six intra-cellular contact points at the end of the four-cell stage (i.e. if any two of the four cells were not in contact with each other) (Liu *et al.*, 2015).

Moreover, the ratios of DC/RC affected blastomeres over total blastomeres in each embryo were analyzed up to Day 3. The <6ICCP was excluded from this analysis as it does not occur at the single blastomere level and is only applicable at the 4-cell stage. Specifically, time-lapse footage was reviewed for DC, RC, or a combination of both, and each embryo was classified into one of five possible ratio groups (0, 0.25, 0.5, 0.75, or 1 as demonstrated in Table 2). For example, if DC or RC occurred at the 1-cell stage, a ratio of 1 was noted and if it occurred in one blastomere at the 2-cell stage, this accounted for a ratio of 0.5. In a 4-cell stage embryo, a ratio of 0.75 could be either a result of three out of the four blastomeres demonstrating DC/RC or a combined effect of one affected blastomere at the 2-cell stage plus one affected blastomere at the 4-cell stage which arose from the non-affected 2-cell stage blastomere. The division of blastomeres at the 4-cell stage was considered up until Day 3.

**Table 2.** Ratios of affected blastomeres over total blastomeres up to the completion of each blastomere division at the 4-cell stage.

Ratio	Scenarios of number of blastomere(s) affected by direct cleavage, reverse cleavage, or mixed at each developmental stage		
	1-cell stage	2-cell stage	4-cell stage
**0**	**0**	**0**	**0**
**0.25**	**–**	**–**	**1**
**0.5**	**–**	**1**	**2**
**0.75**	**–**	**–**	**3**
**1**	**1**	**2**	**4**

### Blastocyst vitrification, warming, and frozen transfer

Blastocysts formed on Day 5 or 6 were graded as per the Gardner system (Gardner and Schoolcraft, 1999) and full blastulation was defined as at least stage 3 in expansion with an A/B grade inner cell mass. Suitable blastocysts were vitrified on Day 5 or 6 immediately after grading. The Rapid-i™ device, RapidVit™, and RapidWarm™ Blast media (Vitrolife) were used for vitrification and warming as per manufacturer’s protocol. A total of 664 resulting blastocysts underwent single frozen transfers (hormone replacement therapy or natural cycles) and were included for live birth and neonatal analysis. All pregnancies were followed up until birth, with the sex of the baby, gestational age and birthweight outcomes being recorded as appropriate.

### Statistical analysis

Continuous data were analyzed via t-test (expressed as mean ± standard deviation) or a multiple linear regression model. Linear regression statistics were expressed as standardized coefficient (*β*) coupled with a 95% confidence interval (CI). Proportional data were evaluated by chi-squared analysis or logistic regression to determine adjusted odds ratios (aOR) with a 95% CI. Statistical analysis was performed using Statistical Package for the Social Sciences (version 25, IBM, Washington, USA) and *P *<* *0.05 was considered as statistically significant.

## Results

### Incidence of abnormal cleavage

**Figure 1.**
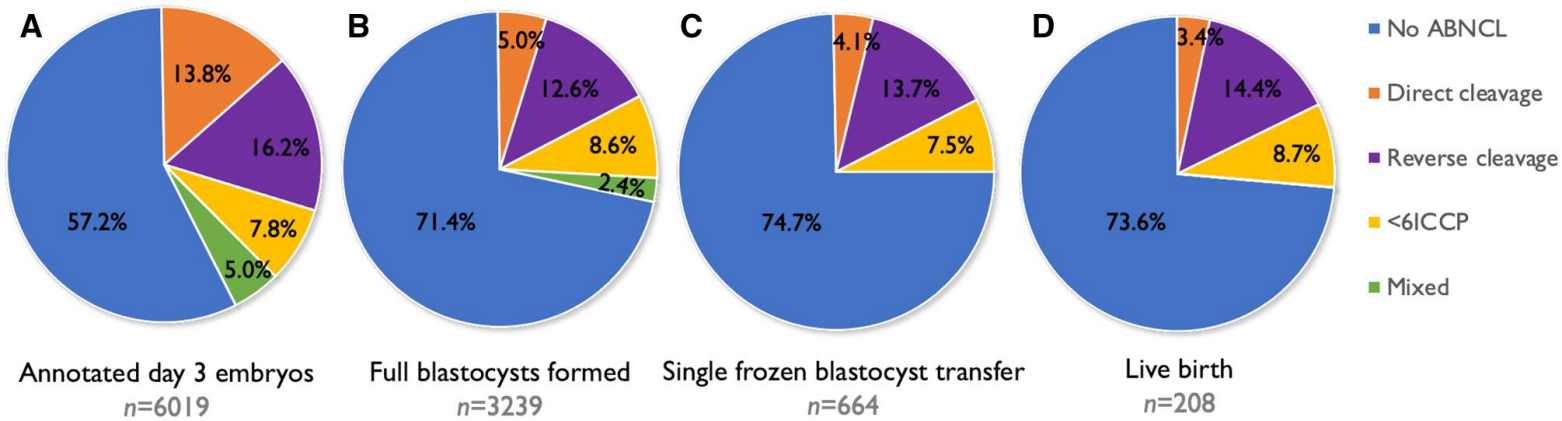
**Incidences of different types of abnormal cleavage between different datasets.** Note: Proportion of embryos showing no abnormal cleavage or abnormal cleavage events in all: (**A**) annotated Day 3 embryos; (**B**) blastocysts formed; (**C**) blastocysts undergoing single frozen transfer; and (**D**) blastocysts leading to a live birth. <6ICCP, less than six intracellular contact points; ABNCL, abnormal cleavage; mixed, embryos showing at least two types of abnormal cleavage patterns.

Out of 6019 fully annotated Day 3 embryos, the majority presented with no ABNCL (57.2%, 3443/6019), followed by RC (16.2%, 976/6019), DC (13.8%, 830/6019), and <6ICCP (7.8%, 471/6019), while 5.0% (299/6019) showed a mixture of two or more ABNCL events (Fig. 1A). In reference to the Day 3 embryo dataset of no ABNCL embryos (57.2%, Fig. 1A), there was a significant difference with the proportion of embryos present in the blastocyst dataset (71.4%, *P *<* *0.01, Fig. 1B), in the transferred blastocyst dataset (74.7%, *P *<* *0.01, Fig. 1C), and in the live birth dataset (73.6%, *P *<* *0.05, Fig. 1D). The compositions of different ABNCL types were similar (*P *>* *0.05) between the transferred blastocysts (Fig. 1C) and live birth datasets (Fig. 1D),

### Full blastulation rate

**Figure 2.**
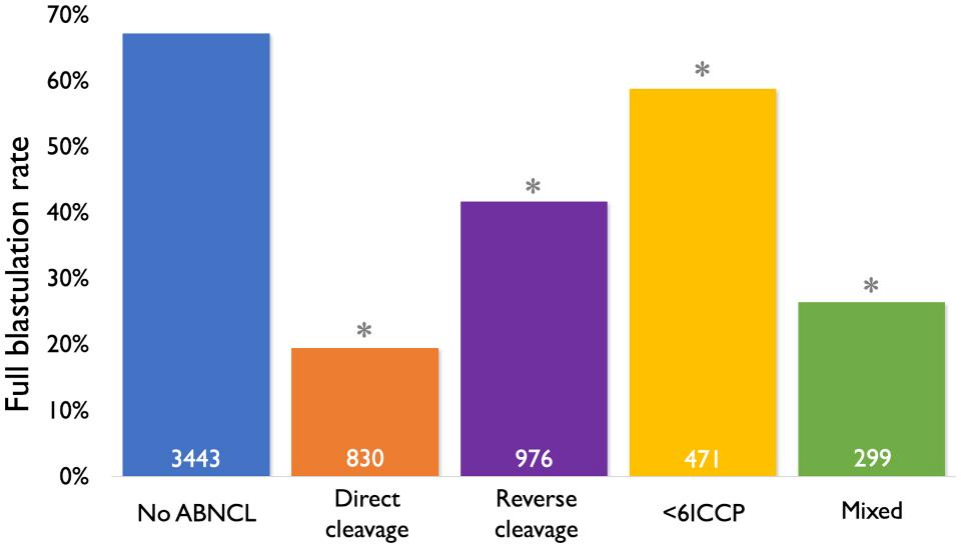
**Blastulation rates of embryos with or without abnormal cleavage events.** ABNCL, abnormal cleavage; <6ICCP, less than six intracellular contact points at the 4-cell stage; mixed, embryos showing at least two types of abnormal cleavage patterns. Numbers within each bar represent the total number of embryos for each group. Statistics and *P* values were based on Chi squared comparisons with the No ABNCL group. *P <* *0.01 compared to no ABNCL.

**Figure 3.**
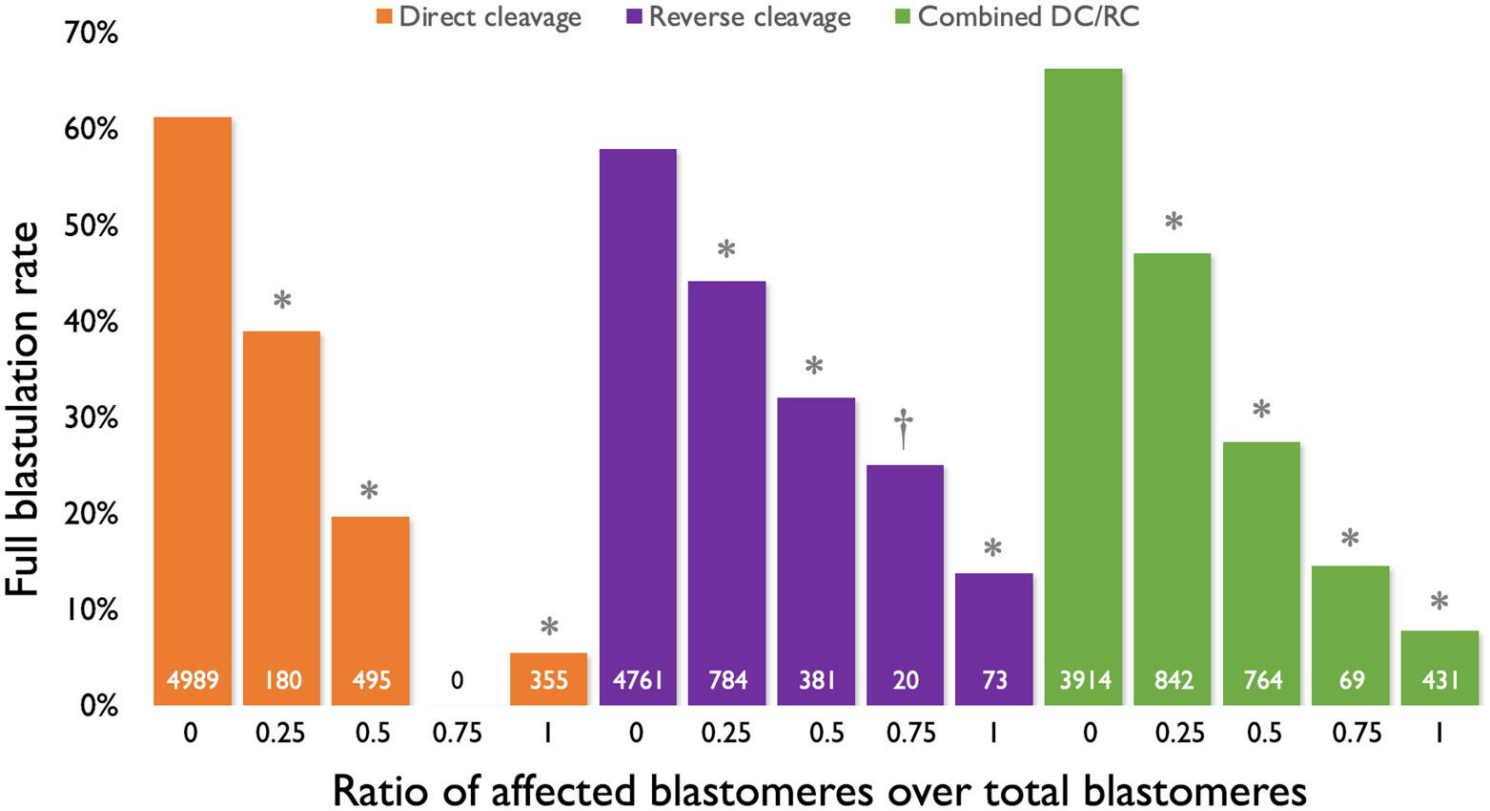
**Blastulation rates of embryos according to ratios of blastomeres affected by direct cleavage, reverse cleavage, or both, over the total blastomeres in the 1- to 8-cell embryos.** DC, direct cleavage; RC, reverse cleavage. Numbers within each bar represent the total number of embryos for each group. Statistics and *P* values were based on Chi squared comparisons with corresponding 0 ratio. *P <* *0.001, ^†^*P *<* *0.05 compared to no affected blastomeres (ratio of 0) within each abnormal cleavage category.

Overall, full blastulation rates for different groups are shown in Fig. 2. Further analysis was performed to investigate the combined impacts of DC/RC on full blastulation based on the ratio of affected blastomeres over all blastomeres. Overall, there was an inverse relationship between the full blastulation rate and the ratio of affected blastomeres over all of the blastomeres (Fig. 3). In the DC group, full blastulation rates were 61.2% (3053/4989), 38.9% (70/180), 19.6% (97/495), 0%, and 5.4% (19/355) for ratios of 0, 0.25, 0.5, 0.75, and 1, respectively; 57.9% (2756/4761), 44.1% (346/784), 32.0% (122/381), 25.0% (5/20), and 13.7% (10/73) for the RC group; and 66.2% (2591/3914), 47.0% (396/842), 27.4% (209/764), 14.5% (10/69), and 7.7% (33/430) in combination of DC and RC groups. All sub-groups of 0.25, 0.5, 0.75, and 1 demonstrated statistical significance compared to their respective 0 ratio group (*P *<* *0.05).

### Live birth rate

A total of 208 single blastocyst transfers resulted in a live birth outcome. There was no significant difference in live birth rates following single frozen transfer of blastocysts with (32.7%) or without ABNCL (30.8%, *P *>* *0.05). Live birth rates were comparable between the DC, RC, <6ICCP, and no ABNCL groups (*P *>* *0.05 respectively) (Table 3). No transfers were performed for the mixed ABNCL group. Logistic regression confirmed no significant impact of DC, RC, or <6ICCP on live birth in comparison to the no ABNCL, after accounting for potential confounders including sequential/single-step culture, maternal age at oocyte collection, female body mass index (BMI), vitrification day, blastocyst expansion stage, inner cell mass/trophectoderm grades, insemination method, and sperm type (partner or donor) (Supplementary Table S1).

**Table 3.** Live birth rate, gestational age, and birthweight following single transfer of blastocysts displaying no abnormal cleavage events (ABNCL), direct cleavage, reverse cleavage, and <6 intercellular contact points at the four-cell stage (ICCP).

	No ABNCL (reference)	Direct cleavage	Reverse cleavage	<6ICCP
**Live birth rate (n* *=* *664)**	30.8% (153/496)	25.9% (7/27)	33.0% (30/91)	36.0% (18/50)
**Gestational age (weeks, n =* *204)**	38.5 ± 1.8	38.7 ± 1.6	38.5 ± 1.2	38.3 ± 3.5
**Birthweight (grams, n* *=* *204)**	3313.9 ± 509.6	3343.0 ± 649.1	3378.2 ± 538.4	3352.6 ± 841.3

Note: Chi-square analysis was performed for live birth rate and *t*-test was performed for gestational age and birthweight. No statistical significance was detected in each abnormal cleavage group compared to the No ABNCL group (*P *>* *0.05).

Further analysis comparing live birth rates of single transferred frozen blastocysts between different ratios of blastomeres affected by DC or RC is shown in Table 4. No significant difference was detected between any categories (*P *>* *0.05).

**Table 4.** Live birth rates according to ratios of blastomeres affected by direct cleavage or reverse cleavage over total blastomeres up to Day 3.

	Live birth rates of single frozen transferred blastocysts according to ratio of blastomeres affected by direct cleavage or reverse cleavage over total blastomeres up to Day 3			
	0.25	0.5	1	Total
**Direct cleavage**	44.4% (4/9)	13.3% (2/15)	33.3% (1/3)	25.9% (7/27)
**Reverse cleavage**	34.3% (23/67)	29.2% (7/24)	—	33.0% (30/91)
**Total**	35.5% (27/76)	23.1% (9/39)	33.3% (1/3)	31.4% (37/118)

Note: No embryos with 75% blastomeres affected were transferred during the study period. No significant difference in live birth rate was detected between each categories (*P *>* *0.05).

### Gestational age and birthweight

Four live birth cycles (out of 208 total live births) were excluded from neonatal analysis: one resulted in monozygotic twins and three had unknown birthweight outcomes. Neonatal outcomes were comparable between the blastocysts with DC, RC, <6ICCP, and no ABNCL for gestational age and birthweight (Table 3).

Multiple linear regression showed no significant impact on the gestational age by DC, RC, and <6ICCP in reference to the no ABNCL group. Similarly, there was no significant impact on birthweight by DC, RC, and <6ICCP in reference to the no ABNCL group. All of the above standard coefficients were adjusted for sequential/single-step culture, maternal age at oocyte collection, female BMI, vitrification day, blastocyst expansion stage, inner cell mass/trophectoderm grades, insemination method, and sperm type (partner or donor) (Supplementary Table S2). Furthermore, no significant difference (*P *>* *0.05, respectively) was detected using either: (i) Z-score combining sex of the baby, birthweight, and gestational age (Supplementary Table S2), (ii) preterm delivery (<37 weeks), or (iii) low birthweight (<2500 g), as separate endpoints (Supplementary Table S1).

## Discussion

This study investigated live birth rates and neonatal outcomes of embryos with or without ABNCL following a single frozen blastocyst transfer. Embryos displaying ABNCL (DC, RC, <6ICCP) by Day 3 had reduced full blastulation rates compared to embryos with no ABNCL. There was a linear relationship between the proportion of blastomeres impacted by ABNCL and full blastulation rates for DC, RC, and a combination of both events. However, once full blastulation was achieved, the live birth rate, gestational age, and birthweight were comparable to embryos with no ABNCL following single frozen blastocyst transfer.

It is widely acknowledged that ABNCL in cleavage stage human embryos leads to poorer clinical outcomes (Rubio *et al.*, 2012; Liu *et al.*, 2014, 2015, 2020). In these studies, the embryos were transferred at the cleavage stage and reduced live birth rates were reported. The observed decrease in live birth rate following Day 3 transfers in other studies could well be explained by a reduced blastulation capability, while embryos failing to blastulate would have been excluded from transfer in a blastocyst transfer program. De-prioritizing ABNCL embryos at transfer seems to be widely adopted in clinical practice, even if they have successfully blastulated (Gorodeckaja *et al.*, 2020). This may have partly contributed to the limited data in the literature, especially for measures such as live birth rate and neonatal outcomes of blastocysts exhibiting ABNCL. In our study, no significant differences were found in live birth rates, gestational age and birthweight when comparing ABNCL blastocysts to those without ABNCL.

In a recent study by Shavit *et al.* (2023), live birth rates and neonatal outcomes of DC embryos were reported using a mixture of single, double and triple embryo transfers. In the study, all fresh transfers were performed on Day 3 with untransferred embryos cultured to the blastocyst stage before cryopreservation. A subset of single frozen blastocyst transfers led to similar live birth rates between the DC and no DC groups following single frozen blastocyst transfer (Shavit *et al.*, 2023). This study is in agreement with our results, though the neonatal results were, however, omitted from their frozen blastocyst transfer analysis. Interestingly, based on a small euploid blastocyst dataset, Ozbek *et al.* (2021) found reduced live birth rates in DC (33.3%, 8/24) and RC blastocysts (23.1%, 6/26) in comparison to the control group (55.9%, 133/238). However, the same study also showed an unexpectedly higher aneuploidy rate in the control blastocysts (57.6%) compared to DC (47.7%) and RC blastocysts (46.2%), implying a potentially biased dataset considering its small sample size. Regardless, our study has shown that there are similar live birth rates and neonatal outcomes despite ABNCL events once full blastulation is achieved. The results of this study are reassuring for counseling patients who are undertaking blastocyst transfers.

Accurate and comprehensive annotations of early developmental events are critical for embryo selection (Lee *et al.*, 2024). Although TLI offers more reliable annotation in comparison to traditional assessment based on static observation (Scott *et al.*, 2021), the increasing number of layers of cells and decreasing blastomere size create challenges for accurate annotation as the embryo develops into later stages. Our study demonstrated that DC/RC can be reliably tracked from the 1-cell up to 8-cell stage (i.e. completion of cell division by each one of the 4-cell blastomeres), enabling annotation of a more complete early cleavage history of the embryo. Reports including annotation at such a level of detail have been scarce in the literature. Barrie *et al.* (2017) reported a comprehensive analysis of a range of ABNCL patterns, including DC, RC Type I and Type II (termed absent cleavage in their study). However, neither blastulation data nor the ratios of affected blastomeres over all blastomeres were analyzed. Shavit *et al.* (2023) reported reduced blastulation when a single early DC event occurred at the first miotic division or multiple DC events occurred during subsequent mitotic divisions, though a single late DC event (up to the third cleavage) did not affect blastulation. This is in general agreement with the findings of our study and others have also reported similar trends (Hlinka *et al.*, 2012; Zhan *et al.*, 2016). By considering the combined impacts of DC/RC up to Day 3, we reported a structured classification system based on the ratios of blastomeres affected over all blastomeres and presented incremental impacts on subsequent blastulation. A full blastulation rate as low as 7.7% was observed when all (ratio of 1) blastomeres were affected by DC/RC, in comparison to 66.2% full blastulation when free from any DC/RC. Similar results were reported by Yang *et al.* (2015) based on a much smaller sample size (n* *=* *345), where a decreasing blastulation rate was identified in embryos with a mixture of several different ABNCL patterns (including DC but without a detailed breakdown analysis) as the cleavage cycles of ABNCL occurrence advanced. However, the authors did not quantify the degree of impact by ABNCL. Such quantitative data presented in our study would potentially facilitate additional selection amongst DC/RC embryos at Day 3 transfer, where extended culture is not possible or offered.

Recently, 3D embryo analysis was utilized to report greater blastocyst development of embryos that had a higher number of cell-contact points at both the 4-cell and 8-cell stages (He *et al.*, 2024). A greater number of contact points at the 8-cell stage was also associated with live birth, but not at the 4-cell stage (He *et al.*, 2024). There is evidence showing planar embryos at the 4-cell stage, a sub-type of <6ICCP under our criteria, is associated with a range of irregular cleavages and decreased blastulation (Ebner *et al.*, 2017). Moreover, Desai and Gill (2019) compared 4-cell embryos with non-tetrahedral blastomere spatial arrangements, considered equivalent to <6ICCP under our criteria, to their tetrahedral counterparts. The authors reported suboptimal blastulation outcomes in the non-tetrahedral group, but similar live birth rates based on 79 single transferred blastocysts with a non-tetrahedral spatial arrangement at the 4-cell stage, which is also in line with our findings. The comparable neonatal outcomes reported in our study offers additional reassurance to patients transferring blastocysts showing a history of <6ICCP at the 4-cell stage.

The association between increasing full blastulation capability and decreasing ratios of DC/RC impact supports a potential self-correction mechanism where embryos autonomously exclude or extrude unwanted blastomere(s) (Coticchio *et al.*, 2021; Parriego *et al.*, 2023). Studies have noted the ejection of abnormal cells resulting from ABNCL blastocysts (Zhan *et al.*, 2016; Lagalla *et al.*, 2017; Ottolini *et al.*, 2017) and there is evidence that a euploid embryo can expel aneuploid blastomeres (Orvieto *et al.*, 2020). It is also hypothesized that cell exclusion or extrusion is a mechanism for aneuploidy rescue and a method of self-correction to keep euploidy status. This notion is supported by the findings of Yang *et al.* (2015), where 71.1% of DC daughter cells did not form part of the blastocyst. Our results warrant further follow-up research tracking participation of DC/RC blastomeres in the compaction and blastulation processes to facilitate better understanding of their corresponding clinical outcomes following transfer. Secondly, alternative self-correction mechanisms are suggested by the findings of our study, where embryos 100% affected by DC/RC are still able to reach full blastulation, albeit at a very low rate (5.4% in DC, 13.7% in RC, and 7.7% combined), and further give rise to a live birth. In our dataset, one out of three transferred blastocysts, that had been 100% affected by DC, achieved a live birth. Unfortunately, our dataset does not include any transferred blastocyst that was 100% affected by RC. Self-correction mechanisms in embryos with 100% blastomeres affected by DC/RC is currently unclear. The underlying mechanism may be shared with single- or multi-pronuclear embryos, backed by emerging evidence showing a reasonable euploidy rate and occasional healthy live birth (Hirata *et al.*, 2020; Bredbacka *et al.*, 2023). Accordingly, further clinical data is required to better understand their true prognosis.

Although the present study’s strengths include a large sample size and consistent annotation of ABNCL over many years, there are limitations that must be addressed. Our study is limited by its retrospective nature as it is difficult to control every known and unknown confounder. Endometrial preparation protocols for frozen embryo transfers were not available in our dataset, although hormone replacement cycles were predominantly used. Some studies have reported different live birth rates with the use of hormone replacement and natural cycles (Zheng *et al.*, 2021; Wang *et al.*, 2023). All ABNCL events were annotated manually, which can have a degree of subjectivity (Lee *et al.*, 2024). There are inter- and intra-operator variations in the annotation of time-lapse measures, including for ABNCL (Sundvall *et al.*, 2013; Adolfsson and Andershed, 2018). Although qualitative measurements like ABNCL are more reproducible than quantitative measurements (Liu *et al.*, 2020), future application of automated annotation of ABNCL events by deep learning would reduce the subjective nature of manual annotation. The wider literature has a range of definitions of DC including rapid cleavage (<5 h for *t*3 − *t*2) (Meseguer *et al.*, 2011; Rubio *et al.*, 2012) and direct uneven cleavage (≤5 h between mother and daughter cell division, or single blastomere directly dividing into three or more blastomeres) (Zhan *et al.*, 2016). The present study considered trichotomous mitosis (single cell dividing directly to three daughter cells at any stage) (Ciray *et al.*, 2014) during annotation and did not consider other types of DC. Furthermore, it is time consuming to build up the live birth dataset for blastocysts involving ABNCL, as they are routinely ranked low priority for transfer in most clinics. Additionally, longitudinal studies of babies born from embryos showing ABNCL are warranted to compare developmental outcomes such as congenital anomalies. Finally, this study was not designed to investigate causal factors of ABNCL occurrence. Two types of culture media were utilized during the study period, and they have been recognized to result in different morphokinetic profiles and the number of good-quality embryos (Hardarson *et al.*, 2015). However, we have controlled for this issue in our multiple logistic regression. Future focused studies are required to enable better understanding of such ABNCL events.

## Conclusion

Our study highlights the important role of blastulation in embryos showing ABNCL up to Day 3, potentially by allowing them to ‘self-correct’. ABNCL embryos have a lower blastocyst formation rate overall; only embryos that self-corrected successfully formed blastocysts. However, once full blastulation has been achieved, ABNCL is not associated with any compromise in subsequent live birth rates, gestational age or birthweight.

## Supplementary Material

deae062_Supplementary_Table_S1

deae062_Supplementary_Table_S2

## Data Availability

The data underlying this article will be shared on reasonable request to the corresponding author.
