# Supplementary material for: Abnormal cleavage up to Day 3 does not compromise live birth and neonatal outcomes of embryos that have achieved full blastulation: a retrospective cohort study
Source: Hum Reprod. 2024 Mar 29;39(5):955–62. doi: 10.1093/humrep/deae062 (PMC11063553; doi:10.1093/humrep/deae062)
Supplement: deae062_Supplementary_Table_S1 [file deae062_supplementary_table_s1.pdf]

OXFORD human reproduction

Human Reproduction, 2024, 1–1
https://doi.org/10.1093/humrep/deae062
Supplementary Data

Supplementary Table S1. Multiple logistic regression analysis investigating impact of abnormal cleavage on live birth and neonatal outcomes.

	Adjusted odds ratios (95% confidence interval)		
	**Direct cleavage**	**Reverse cleavage**	**<6ICCP**
**Live birth (n = 664)**	1.390 (0.523–3.692)	1.669 (0.965–2.887)	1.600 (0.826–3.098)
**Preterm delivery (<37 weeks, n = 204)**	0.769 (0.060–9.844)	0.779 (0.176–3.441)	0.869 (0.165–4.581)
**Low birthweight (<2500 g, *n* = 204)**	–	1.162 (0.164–8.215)	0.878 (0.067–11.540)

Regression analyses were based on blastocysts displaying no abnormal cleavage as reference. Odds ratios were adjusted for sequential/single-step culture, maternal age at egg collection, female body mass index, vitrification day, blastocyst expansion stage, inner cell mass grade, trophectoderm grade, insemination method, and sperm type (partner or donor). No statistical significance was detected in all analyses above ($P > 0.05$). No low birthweight baby was identified in the direct cleavage group. One monozygotic twin pregnancy and three pregnancies with unknown outcomes were excluded from analysis for preterm delivery and low birthweight. <6ICCP, less than six intercellular contact points at the four-cell stage.
