# Supplementary material for: Abnormal cleavage up to Day 3 does not compromise live birth and neonatal outcomes of embryos that have achieved full blastulation: a retrospective cohort study
Source: Hum Reprod. 2024 Mar 29;39(5):955–62. doi: 10.1093/humrep/deae062 (PMC11063553; doi:10.1093/humrep/deae062)
Supplement: deae062_Supplementary_Table_S2 [file deae062_supplementary_table_s2.pdf]

OXFORD
human reproduction

Human Reproduction, 2024, 1–1
https://doi.org/10.1093/humrep/deae062
Supplementary Data

Supplementary Table S2. Multiple linear regression analysis investigating impact of abnormal cleavage on subsequent neonatal outcomes.

	Standardized coefficient (95% confidence interval)		
	**Direct cleavage**	**Reverse cleavage**	**<6ICCP**
**Gestational age (weeks, n = 204)**	0.036 (−1.107–1.875)	0.000 (−0.817–0.821)	−0.033 (−1.188–0.736)
**Birthweight (grams, n = 204)**	0.012 (−395.049–467.307)	0.023 (−200.979–272.229)	0.019 (−241.260–314.675)
**Z-score**	0.018 (−1.300–1.687)	0.010 (−0.767–0.872)	0.062 (−0.544–1.378)

Regression analyses were based on blastocysts displaying no abnormal cleavage as reference. *Z*-score was calculated by incorporating gestational age, birthweight, and sex of the baby. Standard coefficients were adjusted for sequential/single-step culture, maternal age at egg collection, female body mass index, vitrification day, blastocyst expansion stage, inner cell mass grade, trophectoderm grade, insemination method, and sperm type (partner or donor). No statistical significance was detected in all analyses above ($P > 0.05$). <6ICCP, less than six intercellular contact points at the 4-cell stage.
